# Influence of obstetric factors on osteogenic potential of umbilical cord-derived mesenchymal stem cells

**DOI:** 10.1186/1477-7827-7-106

**Published:** 2009-10-05

**Authors:** Letizia Penolazzi, Renata Vecchiatini, Stefania Bignardi, Elisabetta Lambertini, Elena Torreggiani, Alessandro Canella, Tiziana Franceschetti, Giorgio Calura, Fortunato Vesce, Roberta Piva

**Affiliations:** 1 Department of Biochemistry and Molecular Biology, Molecular Biology Section, University of Ferrara, Italy; 2 Department of Medico-Surgical Disciplines of Communication and Behaviour, University of Ferrara, Italy; 3 Department of Biomedical Sciences and Advanced Therapies, Section of Obstetric and Gynaecological Clinic, Azienda Ospedaliero-Universitaria S Anna, Ferrara, Italy; 4 Department of Reconstructive Science, University of Connecticut Health Center, Farmington, Connecticut, USA

## Abstract

Wharton's jelly from the umbilical cord is a noncontroversial source of mesenchymal stem cells (WJMSCs) with high plasticity, proliferation rate and ability to differentiate towards multiple lineages. WJMSCs from different donors have been characterized for their osteogenic potential. Although there is large evidence of WJMSCs plasticity, recently scientific debate has focused on MSCs selection, establishing predictable elements to discriminate the cells with most promising osteoprogenitor cell potential.

In the present study a comparative study between the presence of osteoblastic markers and different parameters that pertain to both the newborn and the mother was performed. Umbilical cords were collected after all patients signed the informed consent and local ethical commettee approved the study. Obstetric parameters, including baby's gender and birth weight, mother's age at delivery, gestational stage at parturition and mode of delivery were examined. After characterization and expansion, WJMSCs were analyzed for two osteoblastic markers, alkaline phosphatase (ALP) activity, and the expression level of RUNX-2 transcription factor, and for their ability to deposit mineralized matrix after osteogenic induction.

We found that osteoblastic potential was not influenced by baby's gender and mode of delivery. On the contrary, the highest degree of osteoblastic potential has been shown by WJMSCs with RUNX-2 high basal levels, selected from umbilical cords of the heaviest term babies.

Even if further evaluation is required, our hypothesis is that our findings may help in selecting the optimal umbilical cord donors and in collecting high potential Wharton's jelly-derived osteoprogenitors efficiently.

## Background

The umbilical cord is a noncontroversial source of mesenchymal stem cells (MSCs) [1,2]. Recently MSCs isolated from Wharton's Jelly (WJMSCs), a mucoid connective tissue of the umbilical cord, were shown to have the ability to differentiate towards multiple lineages, including adipose, bone, and neuronal lineages [3,4].

Work from several laboratories suggests that these cells, which are very abundant, have potential in therapeutic and tissue engineering field, and indicates that they may be successfully collected and stored for both preclinical work, and banking services [5,6].

It is important to point out that patients who receive umbilical cord stem cells are at a lower risk of developing graft versus host disease, than those who receive bone marrow transplants [7].

Even if there is a considerable debate about MSC plasticity, there are numerous recent reviews and papers on MSCs describing molecular signals that have been identified in driving MSC differentiation down osteoblast lineage, and molecules that are known playing an important role in achieving the desired cellular response such as bone morphogenetic proteins (BMP), dexamethasone, ascorbic acid, and β-glycerophosphate [8,9]. Bone defect repair has been one of the first applications of MSCs, and clinical potential of the use of these cells for bone tissue repair is now extensively explored [10-13].

Nevertheless, bone tissue engineering applications require that MSCs must possess certain reproducible characteristics such as maintenance of the differentiated phenotype. In this scenario, an area of intense research activity, is devoted to improve human MSC characterization, isolation, and expansion [14-16].

Starting from these considerations, we sought to establish further elements for selection of the most desirable cell source for obtaining, inside a WJMSCs collection, the cells with most promising ability to differentiate into osteoblasts.

In the present study five different obstetric parameters, including baby's gender and birth weight, mother's age at delivery, gestational stage at parturition and mode of delivery, were correlated with osteoblastic markers, such as ALP activity, RUNX-2 expression and with the ability of WJMSCs to differentiate along osteogenic lineage.

The hypothesis that the correlation among these parameters may help the selection of optimal umbilical cord donors to collect WJMSCs with most promising osteoprogenitor cell potential is discussed.

## Discussion

### Human umbilical cord collection and WJMSCs analysis

Human umbilical cord and umbilical cord blood taken after delivery of the newborn, from samples that would be inevitably discarded, have been regarded as an alternative source for transplantation and therapy because of their haematopoietic and mesenchymal cell components [14]. The increasing interest in mesenchymal progenitors for tissue repair widely promoted the characterization of early predictive parameters for plasticity, inducibility and practical utility of these cells [10].

As previously reported [17] Wharton's jelly is an ideal and uncontroversial source for mesenchymal stem cells (WJMSCs) due to the simple collection procedure and the high homogeneity of cell population which is obtained.

**Figure 1:**
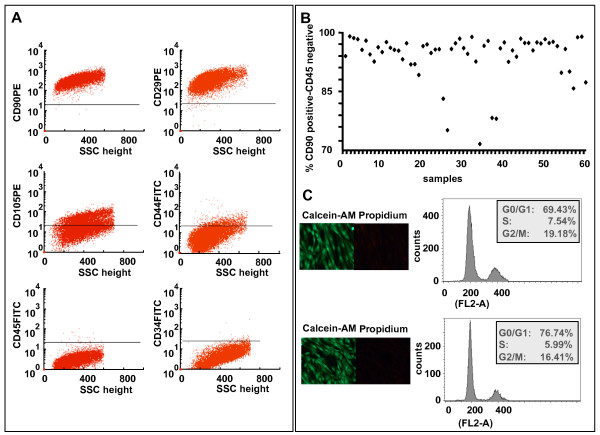
**Small pieces (2-3 mm^2^) of cords were processed within 4 hours and cultured in DMEM-LG**. At ~70-80% confluence, cells were scraped off by 0.05% trypsin/EDTA (Gibco, Grandisland, USA), and analyzed for expression of mesenchymal stem cell surface markers [17], by flow cytometric analysis, as reported (representative experiment) in panel **A**. The gated cells were negative for the hematopoietic line markers CD45 and CD34, partially positive for CD105 and CD44, and positive for the mesenchymal stem cells markers CD90 and CD29. **B**) Schematical distribution of the cell surface parameters of the 60 samples analyzed. **C**) Comparison of the cell culture viability before and after thawing of a cryopreserved sample (high and low panel, respectively). The viability of WJMSCs analyzed by double staining with propidium iodide (PI) and Calcein-AM (Cellstain double staining kit, Sigma Aldrich, St Luis, MO, USA) is indicated. Cells were propidium iodide stained and then analyzed, before and after cryopreservation, for their DNA content, by using BD Immunocytometry Systems DNA QC Particles (BD, New Jersey, USA). The cytofluorimetric profile was analyzed and the percentage of the cell population distribution in the different phases has been reported.

**Figure 2:**
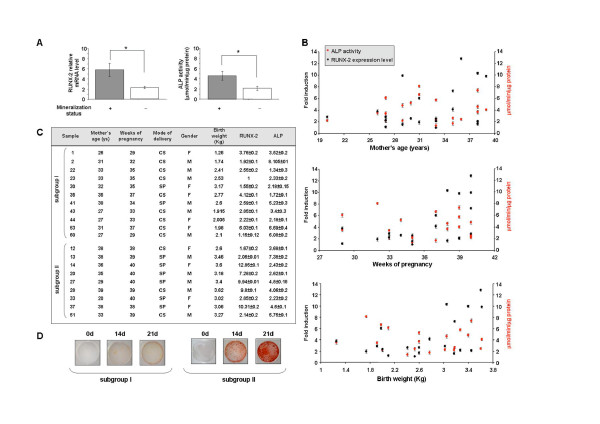
**After characterization in terms of mesenchymal stem cell surface markers expression and cell viability, the osteogenic differentiation of WJMSCs was assessed in the first- and second-passage cultures**. Cells were cultured for 21 days in Osteogenic Differentiation Medium (Osteogenic BulletKit, PT-3924 & PT-4120, Lonza, Basel, Switzerland) or in DMEM-LG as a control. The ability of the cells to become mature osteoblasts was evaluated in terms of mineral matrix deposition assessed by Alizarin Red staining (AR-S, Sigma Aldrich, St Luis, MO, USA). The cells fixed in 70% ethanol for 1 h at room temperature, were washed with PBS, stained with 40 mM AR-S (pH 4.2) for 10 min at room temperature, washed five times with deionized water and incubated in PBS for 15 min to eliminate non-specific staining. The stained matrix was then observed at different magnification using a Leitz microscope. In panel **A **the mineralization status (+, positive or -, negative) was correlated with the basal level (at day ''0'') of RUNX-2 expression and ALP activity. RUNX-2 expression was examined by quantitative TaqMan (ABI PRISM 7700, Applied Biosystems Inc, Foster City, CA, USA) RT-PCR (Assay-on-demand, Hs00231692_m1). The data were normalized on the basis of GAPDH expression and reported as relative mRNA expression levels. ΔΔCt method was used to compare gene expression data. ALP activity was measured by the hydrolysis of p-nitrophenylphosphate (PNPP, Sigma Aldrich, St Luis, MO, USA) [22] and one unit was defined as the amount of enzyme which hydrolyzed 1 μmol/PNPP per minute. Cell protein content was determined according to the Lowry method [23]. (* = P < 0.05). **B**) Relationship between molecular and obstetric parameters. Basal levels of RUNX-2 expression and ALP activity were related to mother's age, weeks of pregnancy and birth weight in 20 WJMSC samples. **C**) The 20 samples of panel **B **were subdived in the two reported subgroups: premature birth (subgroup I) and full term birth (subgroup II). All examined molecular and obstetric parameters are reported. F (female), M (male), CS (Caesarian delivery), SP (spontaneous delivery). **D**) The ability to deposit mineralized matrix was valuated at the indicated times (0, 14, 21 days) in two representative samples of the two subgroups, by Alizarin red staining.

In this study, we prepared primary cultures of WJMSCs from 60 donating subjects whose characteristics are shown in Table 1. Five obstetric factors, including baby's gender and birth weight, mother's age at delivery, gestational stage at parturition and mode of delivery were examined. By flow cytometric analysis (Figure 1A and 1B) and double staining with propidium iodide (PI) and Calcein-AM (Figure 1C) before and after cryopreservation, we demonstrated that all WJMSCs samples showed a comparable mesenchymal property and the same level of viability. These characteristics indicate that the quality of the cells is not influenced by the examined obstetric factors. In order to assess the effect of these maternal and neonatal factors on osteoblastic potential of WJMSCs, we focused on the WJMSCs showing the highest levels of mesenchymal and adhesion markers (CD90/Thy-1, CD29/β-1 integrin, CD44/hyaluronan receptor, and CD105/SH2, endoglin), but not expressing hematopoietic/endothelial markers (CD34 and CD45). 20 samples were induced to osteoblast differentiation (see legend of Figure 2), and the propensity to differentiate into osteoblasts was demonstrated through the different ability of the cells to deposit mineralized matrix. A very high heterogeneity in response to treatment with osteogenic medium was observed, and all attempts to detect a correlation between this ability and one of the examined clinical parameters met not success. It is important to underline that heterogeneity in the behaviour of our WJMSC samples is in agreement with the data obtained by other researchers [18,19] and suggests that a great variability is often present after the recover of these staminal cells.

**Table 1:** Characteristics of subjects

**Sample**	**Mother's age (ys)**	**Weeks of** **pregnancy**	**Mode of delivery**	**Gender**	**Birth weight (Kg)**
1	26	29	CS	F	1.25
2	31	32	CS	M	1.74
3	30	40	CS	F	3
4	30	32	CS	M	1.9
5	30	32	CS	M	1.91
6	37	40	SP	F	3.59
7	21	41	SP	F	3.75
8	35	40	SP	F	3.17
9	33	42	SP	M	3.05
10	31	40	SP	M	3.69
11	38	38	CS	F	3.32
12	38	38	CS	F	2.6
13	38	39	SP	M	3.45
14	36	40	SP	F	3.6
15	29	40	CS	F	3.22
16	33	38	SP	M	3.7
17	34	38	CS	F	3.45
18	35	35	CS	M	3.31
19	34	40	SP	M	3.21
20	35	40	SP	M	3.18
21	32	38	CS	F	3.6
22	33	35	CS	M	2.41
23	33	35	CS	M	2.53
24	40	40	SP	F	3.75
25	38	39	SP	M	3.36
26	29	37	CS	F	3.62
27	29	40	SP	M	3.4
28	39	39	CS	M	3.62
29	33	40	SP	F	3.12
30	32	35	SP	F	3.17
31	29	37	CS	M	2.14
32	37	39	CS	M	3.37
33	20	40	SP	F	3.02
34	41	39	CS	F	2.73
35	35	37	CS	F	2.77
36	34	38	CS	F	2.9
37	38	38	SP	F	3.05
38	34	40	SP	M	3.7
39	32	36	SP	M	2.93
40	27	38	SP	M	3.1
41	30	34	SP	M	2.6
42	31	41	SP	F	3.25
43	27	33	CS	M	1.915
44	27	33	CS	F	2.005
45	26	39	SP	M	3.35
46	34	42	CS	M	3.51
47	23	37	CS	F	2.95
48	30	39	CS	M	4.24
49	33	39	CS	F	3.3
50	37	39	CS	M	2.84
51	33	39	CS	M	3.27
52	32	40	CS	M	3.9
53	31	37	CS	F	1.98
54	35	40	SP	M	3.95
55	23	37	SP	M	3.11
56	40	38	CS	M	2.85
57	37	39	CS	F	3.3
58	25	35	CS	M	3.57
59	25	35	CS	F	2.38
60	27	29	CS	M	2.1

The recorded clinical parameters are: mother's age, weeks of pregnancy, mode of delivery, newborn gender and birth weight.

F (female) M (male) CS (caesarian delivery) SP (spontaneous delivery)

Umbilical cords were collected, after mothers' consent and approval of "Ethical commettee of University of Ferrara" and "Ethical commettee of Sant'Anna Hospital". The study population consisted of 60 healthy pregnant women voluntary enrolled between February 1, 2008 and March 1, 2009.

### WJMSCs and osteoblastogenesis

On the basis of these observations, we conducted further analyses adjusting for two specific markers of osteoblast differentiation: the activity of Alkaline Phosphatase (ALP) [20] and the expression levels of Runt-related transcription factor 2 (RUNX-2) which increases transcription of osteoblast specific genes [17]. As shown in Figure 2A, focusing on basal levels of ALP and RUNX-2, it has been possible to demonstrate that these parameters can be predictive of osteoblastic potential of WJMSCs. In fact, the samples with high basal levels of RUNX-2 and ALP are more prone to deposit mineral matrix if compared to WJMSC with low levels of these two proteins. These observations suggest that it may be possible to discriminate among different WJMSC samples those will have a positive outcome towards osteoblastic differentiation.

### Osteoblastogenesis and clinical parameters

In a next step, we analyzed whether the basal levels of RUNX-2 and ALP correlate with the examined obstetrics factors (Figure 2B). We found that the infant gender and mode of delivery didn't significantly correlate (P > 0.05) with basal RUNX-2 expression and ALP activity. On the other hand, the age of the mother at delivery, has a significant impact on the basal ALP activity but doesn't affect RUNX-2 expression level. Samples collected from mothers which were <32 years old give origin to WJMSCs with high ALP activity. Interestingly, birth weight of the infant was shown to significatively impact on RUNX-2 basal expression level which, as reported in Figure 2B, decreases with the decreasing of the baby's weight. The same relationship was found for the duration of pregnancy. In fact, it was found that WJMSCs from babies born before the 37 weeks of gestation express lower basal level of RUNX-2 than the full term borns. It is very likely that WJMSCs recovered from premature birth contain a high number of undifferentiated cells with high plasticity, a condition which is not actually required for osteoblast differentiation.

As a whole, these findings led us to focus on two parameters, weeks of pregnancy and consequently birth weight of the baby, and RUNX-2 basal levels, subdividing the collected samples in the two subgroups reported in Figure 2C: subgroup I, premature birth with low levels of RUNX-2, and subgroup II, full term birth with high levels of RUNX-2. The ability of the samples belonging to these two subgroups to complete the event of cellular maturation, that is the deposition of mineralized matrix, was then compared. Two representative samples of the two subgroups (Figure 2D) demonstrate that, samples from subgroup I showed a null mineralization status also after 21 day of cell culture in osteogenic medium, whereas samples from subgroup II showed a high level of mineralization beginning from day 14. These findings suggest that maximal WJMSCs osteoblastic potential can be obtained by primary cultures with RUNX-2 high basal levels, selected from the heaviest term babies.

Another clinical observation that is important to do is that the cases below 37 weeks of gestation were all treated with 24 mg of bethametasone two hours before delivery. Such a therapy is routinely given to all the pregnant women delivering prematurely in order to prevent respiratory distress syndrome in the newborns. Therefore, a possible influence of this hormone on stem cells behaviour can be hypothesized. At this regard it has been recently reported that glucocorticoids play an essential role in favouring stem cells differentiation towards adipocyte lineage, thus inhibiting bone and muscle lineages [21]. Such an influence appears to be exerted by inducing myostatin, a potent molecule that regulates muscle development. Therefore, based on the above evidences a possible absence of osteoblastogenesis could have been expected in the samples derived from women delivering prematurely. Since we observed a low or null mineralization status in WJMSCs from such patients, it could be possible to point out a correlation between the two events. However, our analysis showed that this evidence is not always occuring, indicating that the short term high dose of bethametasone administered wasn't ever effective in inhibiting bone lineage.

## Conclusion

To conclude, we should indicate that the analysis of the basal level of RUNX-2 and ALP activity may allow a quick testing of a high number of mesenchymal precursors cultured in vitro and select the more suitable to potentially use for bone tissue engineering application. In addition, for the same aim, our results suggest that it is preferred to recruit the samples from full term borns without paying attention to mother's age.

Even if further evaluation is required, our hypothesis is that our findings may help in selecting the optimal umbilical cord donors and in collecting high potential Wharton's jelly-derived osteoprogenitors efficiently.

## Competing interests

The authors declare that they have no competing interests.

## Authors' contributions

LP partecipated together with RP and TF in the design of the study. LP, RV, ET and AC carried out the experiments. Data analysys was performed by EL, LP, RV and RP. SB and FV collected the samples and performed clinical analysis. The manuscript was written by RP and LP. GC, RV and FV critically read the manuscript. All authors read and approved the final manuscript.
